# Supplementary material for: MethylNet: an automated and modular deep learning approach for DNA methylation analysis
Source: BMC Bioinformatics. 2020 Mar 17;21:108. doi: 10.1186/s12859-020-3443-8 (PMC7076991; doi:10.1186/s12859-020-3443-8)
Supplement: Supplementary file 1 — Additional file 1: Supplementary Table 1. Male to Female Ratio Across Both Datasets. Supplementary Figure 1. Distribution of Age Across Datasets. Supplementary Table 2. Number of Samples for Each Cancer Subtype in Training, Validation, and Test Sets. Supplementary Figure 2. a) Visual flow diagram of method used to find CpG groupings and recapitulation of DNAm profiles. First, the 300 k CpGs are projected into a 6-dimensional embedding using UMAP. Each point in the low dimensional space represents a CpG and proximity between points denotes a shared methylation profile across all of the training samples (n = 503). Then, KMeans clustering was used to find 25 clusters of CpGs with similar profiles. The number of clusters of CpG features were reduced to 8 by filtering out clusters if their variance was above 1 in the 6D space. After that, the CpG features found in each cluster were used to select CpGs to form independent MethylationArrays across the training, validation and test sets. Finally, one autoencoder was trained per each array and the test samples were recapitulated and compared to the original input data; b) Descriptive statistics for final groupings of CpGs and recapitulation scores for each resultant set of CpGs versus the original methylation profiles input into each model. Supplementary Figure 3. Generated/recapitulated beta values versus original beta values for each CpG per individual of the held-out test set (n = 144); b)-f) corresponds to each of eight chosen clusters in order of low to high cluster variance as previously described; a) is an aggregation of the generated/recapitulated versus true beta values of all of the CpG clusters. Supplementary Figure 4. Hierarchically clustered cosine distance matrix between test samples’ VAE-embedded methylation profiles of the held-out test set for the TCGA cohort, colored by: a) Labels assigned to the hierarchical clustering labels for the samples; b) Original TCGA cancer labels; c) RPMM-derived clustering label [file 12859_2020_3443_MOESM1_ESM.docx]

**Supplementary Material**

**Dataset Statistics and Demographics**

Supplementary Table 1: Male to Female Ratio Across Both Datasets

|  | Train | Val | Test |
| --- | --- | --- | --- |
| Pan-Cancer | 2530:2360 | 355:346 | 713:686 |
| Age/Cell-Type | 240:262 | 36:35 | 59:85 |

**
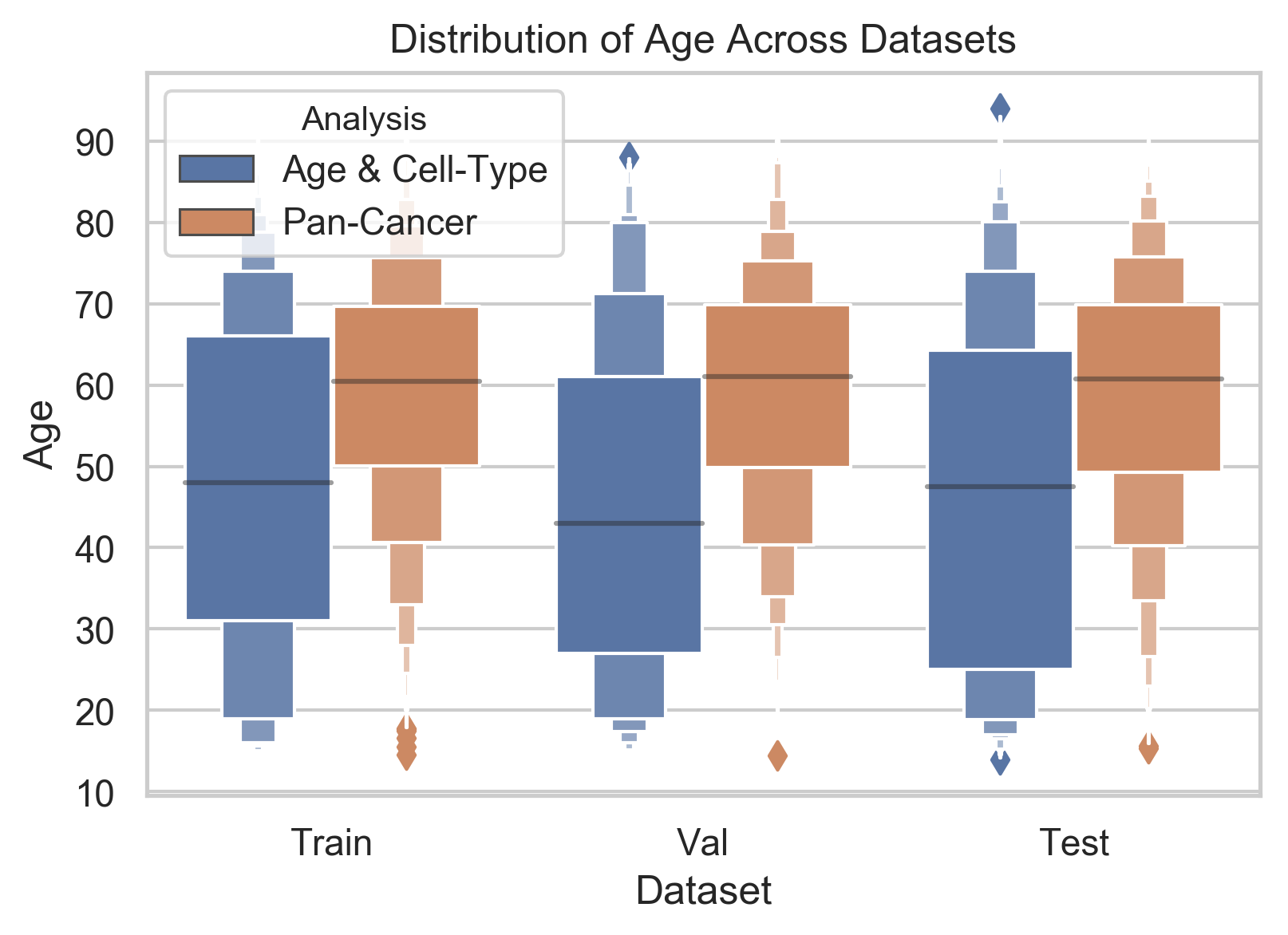
**

**Supplementary Figure 1: Distribution of Age Across Datasets**

Supplementary Table 2: Number of Samples for Each Cancer Subtype in Training, Validation, and Test Sets

|  | Train | Val | Test |
| --- | --- | --- | --- |
| BRCA | 528 | 75 | 151 |
| HNSC | 355 | 51 | 101 |
| LGG | 347 | 50 | 99 |
| THCA | 346 | 50 | 99 |
| PRAD | 341 | 49 | 97 |
| LUAD | 311 | 44 | 89 |
| SKCM | 304 | 44 | 87 |
| UCEC | 298 | 42 | 85 |
| BLCA | 271 | 39 | 77 |
| STAD | 266 | 38 | 76 |
| LIHC | 247 | 35 | 70 |
| LUSC | 247 | 35 | 70 |
| KIRC | 216 | 31 | 62 |
| CESC | 206 | 30 | 59 |
| COAD | 180 | 26 | 52 |
| SARC | 167 | 24 | 48 |
| KIRP | 153 | 22 | 44 |
| LAML | 129 | 18 | 37 |
| PCPG | 122 | 18 | 35 |
| ESCA | 117 | 17 | 34 |
| PAAD | 110 | 16 | 32 |
| GBM | 96 | 14 | 27 |
| TGCT | 93 | 13 | 27 |
| THYM | 84 | 12 | 24 |
| READ | 62 | 9 | 18 |
| MESO | 61 | 9 | 17 |
| ACC | 49 | 7 | 14 |
| UVM | 44 | 6 | 13 |
| KICH | 37 | 5 | 11 |
| DLBC | 32 | 5 | 9 |
| UCS | 32 | 5 | 9 |
| CHOL | 9 | 1 | 3 |

**Unsupervised Analyses – Evaluation of Unsupervised Encoder Performance**

To establish *MethylNet* as a method for DNAm encoding, we first show that it can accurately capture features that dictate DNAm by using the features to recapitulate the supplied DNAm signal. If *MethylNet* can reconstruct the entire methylation profile from its latent-derived features, then those features should have high fidelity to the original dataset.

To support this, we first split up all of the CpGs into groups of features, with an average group size of 14,264 CpGs, that had shared methylation profiles across the training healthy blood samples of the Johansson data (n=503). Then, we fit an autoencoder produced by *MethylNet* to each of the resulting DNAm arrays and proceeded to test the ability to generate synthetic methylation samples on held out test data (Supplementary Figure 2). *MethylNet* was able to recapitulate the beta values with a weighted R^2^-value of 92.6% and weighted mean absolute error of 1.9% methylation between the original and generated held out test set (Supplementary Figures 2,3), demonstrating *MethylNet’s* ability to recapitulate the signal, and encode features with high fidelity to the original dataset.

To further establish encoding performance and provide confidence that future studies could utilize *MethylNet* to assess unknown heterogeneity in methylation profiles associated with disease, we tested whether features processed using the encoder can meaningfully cluster the methylation samples with concordance to known disease subtypes. TCGA DNAm data were encoded using *MethylNet’s* VAE and hierarchical clustering was performed on the embeddings and then compared with clustering results from Recursively Partitioned Mixture Modeling (RPMM) that used 20k CpGs with the highest variance across samples. Cluster labels assigned to the embedded samples demonstrated agreement with the original cancer labels with a score of 0.76, compared to a score of 0.47 using RPMM (Supplementary Figure 4), indicating the potential for VAEs to utilize encoded features discover disease heterogeneity when labels are not supplied.

**Supplementary Figure 2:** a) Visual flow diagram of method used to find CpG groupings and recapitulation of DNAm profiles. First, the 300k CpGs are projected into a 6-dimensional embedding using UMAP. Each point in the low dimensional space represents a CpG and proximity between points denotes a shared methylation profile across all of the training samples (n=503). Then, KMeans clustering was used to find 25 clusters of CpGs with similar profiles. The number of clusters of CpG features were reduced to 8 by filtering out clusters if their variance was above 1 in the 6D space. After that, the CpG features found in each cluster were used to select CpGs to form independent MethylationArrays across the training, validation and test sets. Finally, one autoencoder was trained per each array and the test samples were recapitulated and compared to the original input data; b) Descriptive statistics for final groupings of CpGs and recapitulation scores for each resultant set of CpGs versus the original methylation profiles input into each model.

**Supplementary Figure 3:** Generated/recapitulated beta values versus original beta values for each CpG per individual of the held-out test set (n=144); b)-f) corresponds to each of eight chosen clusters in order of low to high cluster variance as previously described; a) is an aggregation of the generated/recapitulated versus true beta values of all of the CpG clusters

**Supplementary Figure 4:** Hierarchically clustered cosine distance matrix between test samples’ VAE-embedded methylation profiles of the held-out test set for the TCGA cohort, colored by: a) Labels assigned to the hierarchical clustering labels for the samples; b) Original TCGA cancer labels; c) RPMM-derived clustering labels on 20k CpGs. Agreement scores between the RPMM and hierarchal clustering results and the original cancer subtypes were calculated using the v-measure, which takes into account the homogeneity and completeness of the labeling. Note that the clustering colors are not the same because the number of clusters is different from the number of cancer labels.

**Training and Validation Results**

Supplementary Table 3: MethylNet Results on Training (n=503) and Validation (n=72) Sets for Sample Age Prediction

| Dataset | R^2^  (mean±SE) | Mean Absolute Residual  (mean±SE) | Explained Variance Proportion  (mean±SE) |
| --- | --- | --- | --- |
| Training | 0.993±2.57e-04 | 1.49±0.0441 | 0.993±2.45e-04 |
| Validation | 0.973±0.00506 | 2.69±0.247 | 0.973±0.00505 |

Supplementary Table 4: MethylNet Results on Training (n=503) and Validation (n=72) Sets for Cell Type Deconvolution

| Cell Type | Dataset | R^2^  (mean±SE) | Mean Absolute Residual  (mean±SE) | Explained Variance Proportion  (mean±SE) |
| --- | --- | --- | --- | --- |
| B Cell | Train | 0.884±0.00967 | 0.00685±2.4e-04 | 0.89±0.00917 |
|  | Validation | 0.756±0.0374 | 0.0101±9.88e-04 | 0.774±0.0371 |
| CD4T | Train | 0.975±0.00218 | 0.00628±1.79e-04 | 0.988±0.00102 |
|  | Validation | 0.874±0.0288 | 0.0121±0.00115 | 0.89±0.025 |
| CD8T | Train | 0.983±0.00152 | 0.00476±1.66e-04 | 0.986±0.00126 |
|  | Validation | 0.758±0.0469 | 0.0154±0.00149 | 0.771±0.0388 |
| Monocytes | Train | 0.898±0.00947 | 0.00535±1.76e-04 | 0.898±0.00941 |
|  | Validation | 0.385±0.0775 | 0.0122±0.00138 | 0.386±0.076 |
| NK | Train | 0.983±0.00151 | 0.0055±1.7e-04 | 0.99±9.69e-04 |
|  | Validation | 0.819±0.0399 | 0.0121±0.00136 | 0.827±0.0373 |
| Neutrophils | Train | 0.995±4.44e-04 | 0.00524±1.67e-04 | 0.996±3.36e-04 |
|  | Validation | 0.962±0.00942 | 0.012±0.0011 | 0.963±0.00851 |

Supplementary Table 5: MethylNet Results on Training (n=5860) and Validation (n=840) Sets for Pan-Cancer Classification

| Dataset | Accuracy Score | Recall Score | Precision Score | F1-Score |
| --- | --- | --- | --- | --- |
| Train | 1.0±0 | 1.0±0 | 1.0±0 | 1.0±0 |
| Validation | 0.965±0.00622 | 0.965±0.00622 | 0.968±0.00524 | 0.966±0.00607 |

**Differences in Cellular Proportions Identified in VAE Embeddings**

Here, we sought to discover whether unsupervised VAE-derived embeddings could distinguish different cellular proportions to motivate future applications of unsupervised deconvolution methods. The results below indicate that clusters identified from the latent embeddings are able to separate different cellular proportions (save for monocytes) and thus present a possible future opportunity for deconvolution without known references. A total of 33% of hierarchical cluster pairs exhibited differences in B-cell, CD4T and Neutrophil proportions, 13% for CD8T proportions, 7% Natural Killer cellular proportions, and none of the pairs of clusters exhibited differences in Monocyte proportions.

Supplementary Table 6: Tukey’s Studentized Range Tests for identifying which of six hierarchical clusters from unsupervised VAE embeddings differ in cellular proportions; VAE embeddings derived from blood test dataset

| Cell-Type | Cluster 1 | Cluster 2 | Mean Difference Cellular Proportion | Lower | Upper | Reject |
| --- | --- | --- | --- | --- | --- | --- |
| CD4T | 0 | 1 | -0.0593 | -0.0885 | -0.0301 | TRUE |
|  | 0 | 2 | -0.071 | -0.1007 | -0.0413 | TRUE |
|  | 0 | 3 | -0.0686 | -0.1251 | -0.0122 | TRUE |
|  | 0 | 4 | -0.0444 | -0.0769 | -0.012 | TRUE |
|  | 0 | 5 | -0.0739 | -0.1537 | 0.0059 | FALSE |
|  | 1 | 2 | -0.0117 | -0.0237 | 0.0003 | FALSE |
|  | 1 | 3 | -0.0093 | -0.0588 | 0.0401 | FALSE |
|  | 1 | 4 | 0.0149 | -0.0029 | 0.0327 | FALSE |
|  | 1 | 5 | -0.0146 | -0.0896 | 0.0604 | FALSE |
|  | 2 | 3 | 0.0024 | -0.0474 | 0.0521 | FALSE |
|  | 2 | 4 | 0.0266 | 0.0081 | 0.0451 | TRUE |
|  | 2 | 5 | -0.0029 | -0.0781 | 0.0723 | FALSE |
|  | 3 | 4 | 0.0242 | -0.0272 | 0.0756 | FALSE |
|  | 3 | 5 | -0.0053 | -0.0945 | 0.0839 | FALSE |
|  | 4 | 5 | -0.0295 | -0.1058 | 0.0468 | FALSE |
| CD8T | 0 | 1 | -0.0295 | -0.0589 | -0.0001 | TRUE |
|  | 0 | 2 | -0.0238 | -0.0537 | 0.006 | FALSE |
|  | 0 | 3 | -0.0584 | -0.1151 | -0.0016 | TRUE |
|  | 0 | 4 | -0.0148 | -0.0475 | 0.0178 | FALSE |
|  | 0 | 5 | -0.0789 | -0.1592 | 0.0014 | FALSE |
|  | 1 | 2 | 0.0057 | -0.0064 | 0.0177 | FALSE |
|  | 1 | 3 | -0.0289 | -0.0787 | 0.0209 | FALSE |
|  | 1 | 4 | 0.0147 | -0.0032 | 0.0325 | FALSE |
|  | 1 | 5 | -0.0494 | -0.1249 | 0.026 | FALSE |
|  | 2 | 3 | -0.0346 | -0.0846 | 0.0154 | FALSE |
|  | 2 | 4 | 0.009 | -0.0096 | 0.0276 | FALSE |
|  | 2 | 5 | -0.0551 | -0.1307 | 0.0206 | FALSE |
|  | 3 | 4 | 0.0436 | -0.0082 | 0.0953 | FALSE |
|  | 3 | 5 | -0.0205 | -0.1103 | 0.0692 | FALSE |
|  | 4 | 5 | -0.0641 | -0.1409 | 0.0127 | FALSE |
| Bcell | 0 | 1 | -0.0178 | -0.0339 | -0.0018 | TRUE |
|  | 0 | 2 | -0.0278 | -0.0441 | -0.0115 | TRUE |
|  | 0 | 3 | -0.0334 | -0.0644 | -0.0024 | TRUE |
|  | 0 | 4 | -0.0136 | -0.0314 | 0.0042 | FALSE |
|  | 0 | 5 | -0.0352 | -0.079 | 0.0086 | FALSE |
|  | 1 | 2 | -0.01 | -0.0166 | -0.0034 | TRUE |
|  | 1 | 3 | -0.0156 | -0.0427 | 0.0116 | FALSE |
|  | 1 | 4 | 0.0042 | -0.0055 | 0.014 | FALSE |
|  | 1 | 5 | -0.0173 | -0.0585 | 0.0238 | FALSE |
|  | 2 | 3 | -0.0056 | -0.0329 | 0.0217 | FALSE |
|  | 2 | 4 | 0.0142 | 0.0041 | 0.0244 | TRUE |
|  | 2 | 5 | -0.0073 | -0.0486 | 0.0339 | FALSE |
|  | 3 | 4 | 0.0198 | -0.0084 | 0.048 | FALSE |
|  | 3 | 5 | -0.0018 | -0.0507 | 0.0472 | FALSE |
|  | 4 | 5 | -0.0216 | -0.0635 | 0.0203 | FALSE |
| Mono | 0 | 1 | 0.0049 | -0.0088 | 0.0186 | FALSE |
|  | 0 | 2 | 0.0041 | -0.0098 | 0.018 | FALSE |
|  | 0 | 3 | 0.0058 | -0.0206 | 0.0323 | FALSE |
|  | 0 | 4 | 0.0017 | -0.0135 | 0.0169 | FALSE |
|  | 0 | 5 | 0.0051 | -0.0323 | 0.0425 | FALSE |
|  | 1 | 2 | -0.0008 | -0.0065 | 0.0048 | FALSE |
|  | 1 | 3 | 0.0009 | -0.0222 | 0.0241 | FALSE |
|  | 1 | 4 | -0.0032 | -0.0115 | 0.0052 | FALSE |
|  | 1 | 5 | 0.0002 | -0.0349 | 0.0354 | FALSE |
|  | 2 | 3 | 0.0018 | -0.0215 | 0.0251 | FALSE |
|  | 2 | 4 | -0.0023 | -0.011 | 0.0063 | FALSE |
|  | 2 | 5 | 0.0011 | -0.0342 | 0.0363 | FALSE |
|  | 3 | 4 | -0.0041 | -0.0282 | 0.02 | FALSE |
|  | 3 | 5 | -0.0007 | -0.0425 | 0.0411 | FALSE |
|  | 4 | 5 | 0.0034 | -0.0324 | 0.0392 | FALSE |
| NK | 0 | 1 | -0.0107 | -0.0431 | 0.0217 | FALSE |
|  | 0 | 2 | -0.0199 | -0.0528 | 0.013 | FALSE |
|  | 0 | 3 | -0.0127 | -0.0753 | 0.0498 | FALSE |
|  | 0 | 4 | -0.035 | -0.071 | 0.001 | FALSE |
|  | 0 | 5 | -0.0395 | -0.128 | 0.0489 | FALSE |
|  | 1 | 2 | -0.0092 | -0.0225 | 0.0041 | FALSE |
|  | 1 | 3 | -0.0021 | -0.0569 | 0.0528 | FALSE |
|  | 1 | 4 | -0.0243 | -0.044 | -0.0046 | TRUE |
|  | 1 | 5 | -0.0289 | -0.112 | 0.0543 | FALSE |
|  | 2 | 3 | 0.0072 | -0.0479 | 0.0623 | FALSE |
|  | 2 | 4 | -0.0151 | -0.0356 | 0.0054 | FALSE |
|  | 2 | 5 | -0.0196 | -0.103 | 0.0637 | FALSE |
|  | 3 | 4 | -0.0223 | -0.0793 | 0.0347 | FALSE |
|  | 3 | 5 | -0.0268 | -0.1257 | 0.0721 | FALSE |
|  | 4 | 5 | -0.0045 | -0.0892 | 0.0801 | FALSE |
| Neu | 0 | 1 | 0.0919 | 0.0356 | 0.1482 | TRUE |
|  | 0 | 2 | 0.1029 | 0.0458 | 0.16 | TRUE |
|  | 0 | 3 | 0.1281 | 0.0195 | 0.2368 | TRUE |
|  | 0 | 4 | 0.0799 | 0.0175 | 0.1424 | TRUE |
|  | 0 | 5 | 0.1808 | 0.0272 | 0.3344 | TRUE |
|  | 1 | 2 | 0.011 | -0.0121 | 0.0341 | FALSE |
|  | 1 | 3 | 0.0362 | -0.059 | 0.1314 | FALSE |
|  | 1 | 4 | -0.012 | -0.0462 | 0.0223 | FALSE |
|  | 1 | 5 | 0.0889 | -0.0556 | 0.2334 | FALSE |
|  | 2 | 3 | 0.0252 | -0.0705 | 0.121 | FALSE |
|  | 2 | 4 | -0.023 | -0.0586 | 0.0126 | FALSE |
|  | 2 | 5 | 0.0779 | -0.0669 | 0.2227 | FALSE |
|  | 3 | 4 | -0.0482 | -0.1472 | 0.0508 | FALSE |
|  | 3 | 5 | 0.0527 | -0.1191 | 0.2244 | FALSE |
|  | 4 | 5 | 0.1009 | -0.0461 | 0.2479 | FALSE |

**Comparison of MethylNet Cellular Proportions to Robust Partial Corrections and Cibersort**

Here, we report the concordance the results obtained using MethylNet for cellular proportions to the other estimation methods, but highlight that the choice of estimateCellCounts2 as a ground truth is based on our previously published results showing that is has significantly higher accuracy compared with MethylCiBERSort and RPC when flow cytometry measurements are set ground truth (Salas LA, Genome Biology, 2018):

Supplementary Table 7: Correlation of MethylNet Cellular Proportions to Other Estimation Methods’ Proportions

| Cell Type | Robust Partial Correlations | | Cibersort | |
| --- | --- | --- | --- | --- |
|  | Pearson-R | P-Value | Pearson-R | P-Value |
| CD8T | 0.78 | 1.77E-30 | 0.76 | 1.97E-28 |
| CD4T | 0.89 | 5.08E-49 | 0.87 | 4.85E-45 |
| NK | 0.56 | 3.33E-13 | 0.53 | 1.42E-11 |
| Bcell | 0.84 | 3.60E-39 | 0.84 | 7.50E-39 |
| Mono | 0.38 | 2.07E-06 | 0.33 | 4.17E-05 |
| Neu | 0.97 | 3.30E-94 | 0.97 | 2.44E-92 |

**SHAP Overlap with IDOL**

**
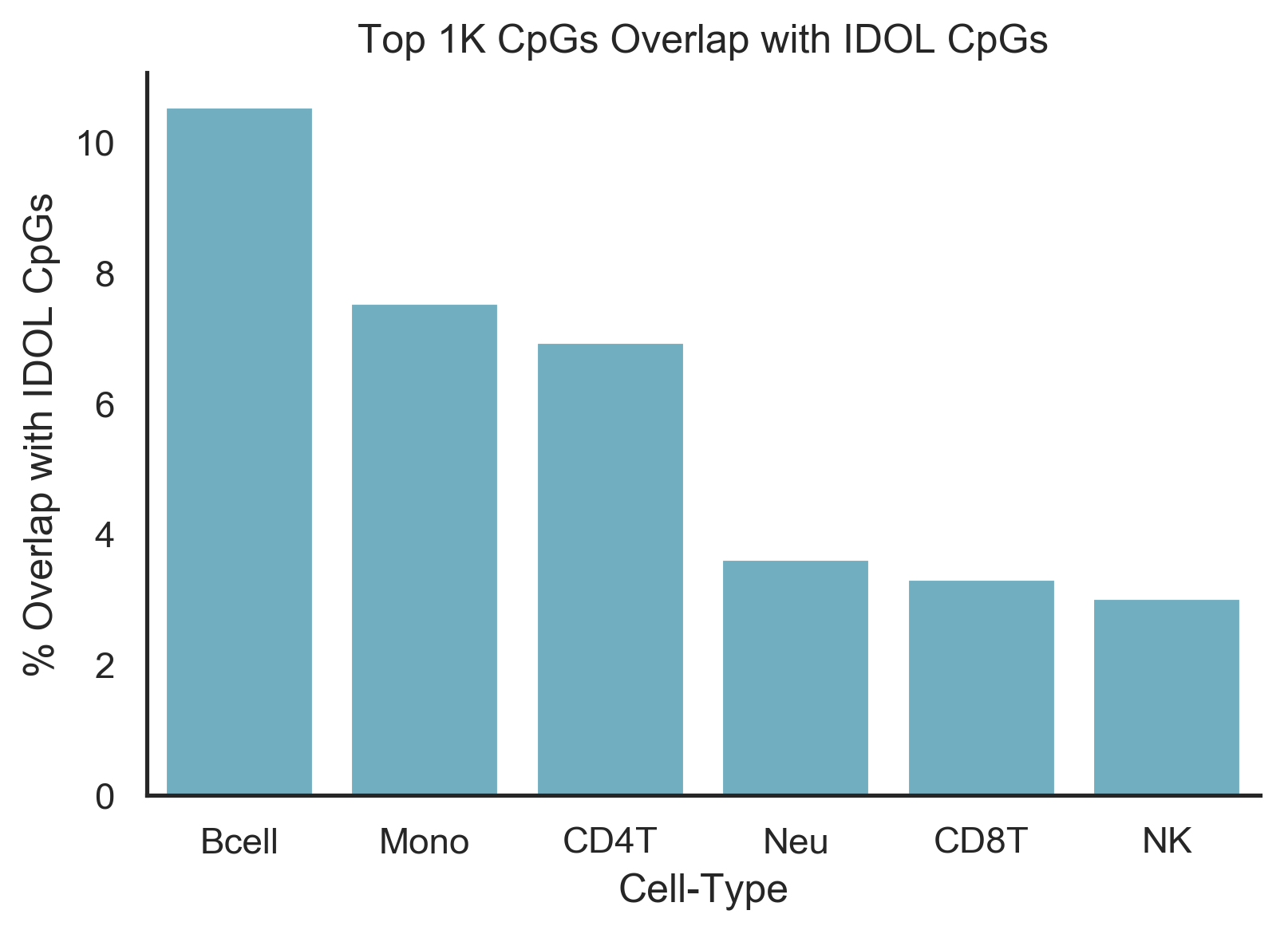
**

**Supplementary Figure 5:** Proportion of IDOL CpGs that are Overlapped by the Top 1k CpGs for Each Cell-Type

**Select Distributions of SHAPley Scores across Age and Cell-Type Groupings**

**Supplementary Figure 6:** Bar Charts of CpGs with the 10 Largest Shapley Scores for Each Cell-Type, linked by red, blue or green lines if shared across subtype for: a-d) Lymphocytes; e-f) Myeloids. Not sharing top 10 CpGs does not indicate that two cell-types do not share similar CpG profiles.

**Supplementary Figure 7:** Bar Charts of CpGs with the 10 Largest Shapley Scores for Age Groups: a) 14-24 and b) 84-94. These CpGs are linked if shared across the age groups, but this does not indicate that they are not shared outside of this top 10 list of CpGs. The top 10 CpGs that are associated with lower age are similar to the older age group; c) Distribution of Shapley Scores for these two age groups. CpG contributions tend to be negative for the younger age groups and positive for the older age groups.

**Confusion Matrix Pan-Cancer Classifications**

Supplementary Table 8: Confusion Matrix Pan-cancer Classification (Colored Superclass)

|  |  | **True Subtypes** | | | | | | | | | | | | | | | | | | | | | | | | | | | | | | | |
| --- | --- | --- | --- | --- | --- | --- | --- | --- | --- | --- | --- | --- | --- | --- | --- | --- | --- | --- | --- | --- | --- | --- | --- | --- | --- | --- | --- | --- | --- | --- | --- | --- | --- |
|  |  | **SARC** | **SKCM** | **UVM** | **BRCA** | **CHOL** | **LIHC** | **KICH** | **KIRC** | **KIRP** | **BLCA** | **CESC** | **UCEC** | **UCS** | **COAD** | **READ** | **LAML** | **ACC** | **PCPG** | **LUAD** | **MESO** | **THCA** | **ESCA** | **STAD** | **PAAD** | **PRAD** | **GBM** | **LGG** | **TGCT** | **THYM** | **HNSC** | **DLBC** | **LUSC** |
| **Predicted Subtypes** | **SARC** | **45** | 0 | 0 | 0 | 0 | 0 | 0 | 0 | 0 | **1** | 0 | 0 | 0 | 0 | 0 | 0 | 0 | 0 | 0 | 2 | 0 | 0 | 0 | 0 | 0 | **1** | 0 | 0 | 0 | 0 | 0 | 0 |
|  | **SKCM** | **1** | **87** | 0 | **1** | 0 | 0 | 0 | 0 | 0 | 0 | 0 | 0 | 0 | 0 | 0 | 0 | 0 | 0 | 0 | 0 | 0 | 0 | 0 | 0 | 0 | 0 | 0 | 0 | 0 | 0 | 0 | 0 |
|  | **UVM** | 0 | 0 | **13** | 0 | 0 | 0 | 0 | 0 | 0 | 0 | 0 | 0 | 0 | 0 | 0 | 0 | 0 | 0 | 0 | 0 | 0 | 0 | 0 | 0 | 0 | 0 | 0 | 0 | 0 | 0 | 0 | 0 |
|  | **BRCA** | **1** | 0 | 0 | **147** | 0 | 0 | 0 | 0 | 0 | 0 | 0 | 0 | 0 | 0 | 0 | 0 | 0 | 0 | 0 | 0 | 0 | 0 | 0 | 0 | 0 | 0 | 0 | 0 | 0 | **1** | 0 | 0 |
|  | **CHOL** | 0 | 0 | 0 | 0 | **3** | **1** | 0 | 0 | 0 | 0 | 0 | 0 | 0 | 0 | 0 | 0 | 0 | 0 | 0 | 0 | 0 | 0 | 0 | 0 | 0 | 0 | 0 | 0 | 0 | 0 | 0 | 0 |
|  | **LIHC** | 0 | 0 | 0 | 0 | 0 | **69** | 0 | 0 | 0 | 0 | 0 | 0 | 0 | 0 | 0 | 0 | 0 | 0 | 0 | 0 | 0 | 0 | 0 | 0 | 0 | 0 | 0 | 0 | 0 | 0 | 0 | 0 |
|  | **KICH** | 0 | 0 | 0 | 0 | 0 | 0 | **11** | 0 | 0 | 0 | 0 | 0 | 0 | 0 | 0 | 0 | 0 | 0 | 0 | 0 | 0 | 0 | 0 | 0 | 0 | 0 | 0 | 0 | 0 | 0 | 0 | 0 |
|  | **KIRC** | 0 | 0 | 0 | 0 | 0 | 0 | 0 | **60** | **2** | 0 | 0 | 0 | 0 | 0 | 0 | 0 | 0 | 0 | 0 | 0 | 0 | 0 | 0 | 0 | 0 | 0 | 0 | 0 | 0 | 0 | 0 | 0 |
|  | **KIRP** | 0 | 0 | 0 | 0 | 0 | 0 | 0 | **2** | **41** | 0 | 0 | 0 | 0 | 0 | 0 | 0 | 0 | 0 | 0 | 0 | 0 | 0 | 0 | 0 | 0 | 0 | 0 | 0 | 0 | 0 | 0 | 0 |
|  | **BLCA** | 0 | 0 | 0 | 0 | 0 | 0 | 0 | 0 | **1** | **75** | 0 | 0 | 0 | 0 | 0 | 0 | 0 | 0 | 0 | 0 | 0 | 0 | 0 | 0 | 0 | 0 | 0 | 0 | 0 | 0 | 0 | 0 |
|  | **CESC** | 0 | 0 | 0 | 0 | 0 | 0 | 0 | 0 | 0 | **1** | **57** | 0 | 0 | 0 | 0 | 0 | 0 | 0 | 0 | 0 | 0 | 0 | 0 | 0 | 0 | 0 | 0 | 0 | 0 | **1** | 0 | 0 |
|  | **UCEC** | **1** | 0 | 0 | 0 | 0 | 0 | 0 | 0 | 0 | 0 | **2** | **84** | 0 | 0 | 0 | 0 | 0 | 0 | 0 | 0 | 0 | 0 | 0 | 0 | 0 | 0 | 0 | 0 | 0 | 0 | 0 | 0 |
|  | **UCS** | 0 | 0 | 0 | **1** | 0 | 0 | 0 | 0 | 0 | 0 | 0 | 0 | **9** | 0 | 0 | 0 | 0 | 0 | **1** | 0 | 0 | 0 | 0 | 0 | 0 | 0 | 0 | 0 | 0 | 0 | 0 | 0 |
|  | **COAD** | 0 | 0 | 0 | 0 | 0 | 0 | 0 | 0 | 0 | 0 | 0 | 0 | 0 | **49** | **2** | 0 | 0 | 0 | 0 | 0 | 0 | 0 | 0 | **1** | 0 | 0 | 0 | 0 | 0 | 0 | 0 | 0 |
|  | **READ** | 0 | 0 | 0 | 0 | 0 | 0 | 0 | 0 | 0 | 0 | 0 | 0 | 0 | **3** | **16** | 0 | 0 | 0 | 0 | 0 | 0 | 0 | 0 | **1** | 0 | 0 | 0 | 0 | 0 | 0 | 0 | 0 |
|  | **LAML** | 0 | 0 | 0 | 0 | 0 | 0 | 0 | 0 | 0 | 0 | 0 | 0 | 0 | 0 | 0 | **37** | 0 | 0 | 0 | 0 | 0 | 0 | 0 | 0 | 0 | 0 | 0 | 0 | 0 | 0 | 0 | 0 |
|  | **ACC** | 0 | 0 | 0 | 0 | 0 | 0 | 0 | 0 | 0 | 0 | 0 | 0 | 0 | 0 | 0 | 0 | **14** | 0 | 0 | 0 | 0 | 0 | 0 | 0 | 0 | 0 | 0 | 0 | 0 | 0 | 0 | 0 |
|  | **PCPG** | 0 | 0 | 0 | 0 | 0 | 0 | 0 | 0 | 0 | 0 | 0 | 0 | 0 | 0 | 0 | 0 | 0 | **34** | 0 | 0 | 0 | 0 | 0 | 0 | 0 | 0 | 0 | 0 | 0 | 0 | 0 | 0 |
|  | **LUAD** | 0 | 0 | 0 | 0 | 0 | 0 | 0 | 0 | 0 | 0 | 0 | 0 | 0 | 0 | 0 | 0 | 0 | 0 | **87** | 0 | 0 | 0 | 0 | 0 | 0 | 0 | 0 | 0 | 0 | 0 | 0 | **4** |
|  | **MESO** | 0 | 0 | 0 | **1** | 0 | 0 | 0 | 0 | 0 | 0 | 0 | **1** | 0 | 0 | 0 | 0 | 0 | 0 | 0 | **15** | 0 | 0 | 0 | **1** | 0 | 0 | 0 | 0 | 0 | 0 | 0 | 0 |
|  | **THCA** | 0 | 0 | 0 | 0 | 0 | 0 | 0 | 0 | 0 | 0 | 0 | 0 | 0 | 0 | 0 | 0 | 0 | 0 | 0 | 0 | **99** | 0 | 0 | 0 | 0 | 0 | 0 | 0 | 0 | **1** | 0 | 0 |
|  | **ESCA** | 0 | 0 | 0 | 0 | 0 | 0 | 0 | 0 | 0 | 0 | 0 | 0 | 0 | 0 | 0 | 0 | 0 | 0 | 0 | 0 | 0 | **29** | **3** | 0 | 0 | 0 | 0 | 0 | 0 | **1** | 0 | 0 |
|  | **STAD** | 0 | 0 | 0 | 0 | 0 | 0 | 0 | 0 | 0 | 0 | 0 | 0 | 0 | 0 | 0 | 0 | 0 | 0 | 0 | 0 | 0 | **4** | **72** | 0 | 0 | 0 | 0 | 0 | 0 | 0 | 0 | 0 |
|  | **PAAD** | 0 | 0 | 0 | 0 | 0 | 0 | 0 | 0 | 0 | 0 | 0 | 0 | 0 | 0 | 0 | 0 | 0 | **1** | 0 | 0 | 0 | 0 | 0 | **29** | 0 | 0 | 0 | 0 | 0 | 0 | 0 | 0 |
|  | **PRAD** | 0 | 0 | 0 | 0 | 0 | 0 | 0 | 0 | 0 | 0 | 0 | 0 | 0 | 0 | 0 | 0 | 0 | 0 | 0 | 0 | 0 | 0 | 0 | 0 | **97** | 0 | 0 | 0 | 0 | 0 | 0 | 0 |
|  | **GBM** | 0 | 0 | 0 | 0 | 0 | 0 | 0 | 0 | 0 | 0 | 0 | 0 | 0 | 0 | 0 | 0 | 0 | 0 | 0 | 0 | 0 | 0 | 0 | 0 | 0 | **25** | **7** | 0 | 0 | 0 | 0 | 0 |
|  | **LGG** | 0 | 0 | 0 | **1** | 0 | 0 | 0 | 0 | 0 | 0 | 0 | 0 | 0 | 0 | 0 | 0 | 0 | 0 | 0 | 0 | 0 | 0 | 0 | 0 | 0 | **1** | **92** | 0 | 0 | 0 | 0 | 0 |
|  | **TGCT** | 0 | 0 | 0 | 0 | 0 | 0 | 0 | 0 | 0 | 0 | 0 | 0 | 0 | 0 | 0 | 0 | 0 | 0 | 0 | 0 | 0 | 0 | 0 | 0 | 0 | 0 | 0 | **27** | 0 | 0 | 0 | 0 |
|  | **THYM** | 0 | 0 | 0 | 0 | 0 | 0 | 0 | 0 | 0 | 0 | 0 | 0 | 0 | 0 | 0 | 0 | 0 | 0 | 0 | 0 | 0 | 0 | 0 | 0 | 0 | 0 | 0 | 0 | **24** | 0 | 0 | 0 |
|  | **HNSC** | 0 | 0 | 0 | 0 | 0 | 0 | 0 | 0 | 0 | 0 | 0 | 0 | 0 | 0 | 0 | 0 | 0 | 0 | 0 | 0 | 0 | **1** | 0 | 0 | 0 | 0 | 0 | 0 | 0 | **97** | 0 | **1** |
|  | **DLBC** | 0 | 0 | 0 | 0 | 0 | 0 | 0 | 0 | 0 | 0 | 0 | 0 | 0 | 0 | 0 | 0 | 0 | 0 | 0 | 0 | 0 | 0 | 0 | 0 | 0 | 0 | 0 | 0 | 0 | 0 | **9** | 0 |
|  | **LUSC** | 0 | 0 | 0 | 0 | 0 | 0 | 0 | 0 | 0 | 0 | 0 | 0 | 0 | 0 | 0 | 0 | 0 | 0 | **1** | 0 | 0 | 0 | **1** | 0 | 0 | 0 | 0 | 0 | 0 | 0 | 0 | **65** |

**Breakdown of Pan-cancer Classifications**

| Supplementary Table 9: Breakdown Pancancer Classification Results (Colored by Superclass) | | |
| --- | --- | --- |
|  | Accuracy-Score | F1-Score |
| SARC | 0.94 | 0.97 |
| SKCM | 1.00 | 1.00 |
| UVM | 1.00 | 1.00 |
| BRCA | 0.97 | 0.99 |
| CHOL | 1.00 | 1.00 |
| LIHC | 0.99 | 0.99 |
| KICH | 1.00 | 1.00 |
| KIRC | 0.97 | 0.98 |
| KIRP | 0.93 | 0.96 |
| BLCA | 0.97 | 0.99 |
| CESC | 0.97 | 0.98 |
| UCEC | 0.99 | 0.99 |
| UCS | 1.00 | 1.00 |
| COAD | 0.94 | 0.97 |
| READ | 0.89 | 0.94 |
| LAML | 1.00 | 1.00 |
| ACC | 1.00 | 1.00 |
| PCPG | 0.97 | 0.99 |
| LUAD | 0.98 | 0.99 |
| MESO | 0.88 | 0.94 |
| THCA | 1.00 | 1.00 |
| ESCA | 0.85 | 0.92 |
| STAD | 0.95 | 0.97 |
| PAAD | 0.91 | 0.95 |
| PRAD | 1.00 | 1.00 |
| GBM | 0.93 | 0.96 |
| LGG | 0.93 | 0.96 |
| TGCT | 1.00 | 1.00 |
| THYM | 1.00 | 1.00 |
| HNSC | 0.96 | 0.98 |
| DLBC | 1.00 | 1.00 |
| LUSC | 0.93 | 0.96 |

**Average Cosine Distance Matrix between Cancer Subtypes for Pancancer-Embeddings**

Supplementary Table 10: Average Cosine Distance Between Embeddings of Cancer Subtypes

| **Subtypes** | | | | | | | | | | | | | | | | | | | | | | | | | | | | | | | | | |  |
| --- | --- | --- | --- | --- | --- | --- | --- | --- | --- | --- | --- | --- | --- | --- | --- | --- | --- | --- | --- | --- | --- | --- | --- | --- | --- | --- | --- | --- | --- | --- | --- | --- | --- | --- |
|  | | **SARC** | **SKCM** | **UVM** | **BRCA** | **CHOL** | **LIHC** | **KICH** | **KIRC** | **KIRP** | **BLCA** | **CESC** | **UCEC** | **UCS** | **COAD** | **READ** | **LAML** | **ACC** | **PCPG** | **LUAD** | **MESO** | **THCA** | **ESCA** | **STAD** | **PAAD** | **PRAD** | **GBM** | **LGG** | **TGCT** | **THYM** | **HNSC** | **DLBC** | **LUSC** | |
| **Subtypes** | **SARC** | 0.0 | 0.7 | 0.7 | 1.0 | 0.8 | 1.0 | 0.7 | 0.9 | 1.0 | 1.0 | 0.8 | 0.8 | 0.7 | 1.2 | 0.9 | 0.9 | 0.8 | 0.9 | 1.4 | 0.6 | 0.9 | 0.9 | 0.8 | 0.7 | 1.2 | 0.8 | 0.9 | 0.9 | 0.9 | 0.9 | 0.7 | 1.1 | |
|  | **SKCM** | 0.7 | 0.0 | 0.6 | 0.9 | 1.0 | 0.8 | 1.0 | 1.0 | 0.9 | 1.1 | 1.2 | 0.8 | 0.8 | 1.1 | 1.3 | 0.7 | 0.8 | 0.9 | 0.9 | 0.8 | 0.8 | 1.2 | 1.1 | 1.3 | 1.7 | 1.0 | 0.7 | 0.9 | 0.9 | 1.0 | 0.8 | 1.0 | |
|  | **UVM** | 0.7 | 0.6 | 0.0 | 0.8 | 0.9 | 0.7 | 1.2 | 0.7 | 0.8 | 1.6 | 1.2 | 0.8 | 1.3 | 0.9 | 1.0 | 0.7 | 1.0 | 0.7 | 1.4 | 1.0 | 1.0 | 1.2 | 1.3 | 1.2 | 1.4 | 1.1 | 0.8 | 1.0 | 0.7 | 1.0 | 1.0 | 1.5 | |
|  | **BRCA** | 1.0 | 0.9 | 0.8 | 0.0 | 0.8 | 0.9 | 1.3 | 1.5 | 1.2 | 1.3 | 0.9 | 0.9 | 0.9 | 0.9 | 1.1 | 1.1 | 0.9 | 0.9 | 0.9 | 0.8 | 1.3 | 0.9 | 1.1 | 1.1 | 1.1 | 1.4 | 1.0 | 1.0 | 0.8 | 1.2 | 1.4 | 1.3 | |
|  | **CHOL** | 0.8 | 1.0 | 0.9 | 0.8 | 0.0 | 0.3 | 0.7 | 1.0 | 0.9 | 1.1 | 0.6 | 0.9 | 1.0 | 1.1 | 0.9 | 1.3 | 1.0 | 1.3 | 0.9 | 0.7 | 0.7 | 0.9 | 0.8 | 0.8 | 0.9 | 1.4 | 1.2 | 1.2 | 0.9 | 1.3 | 0.7 | 1.0 | |
|  | **LIHC** | 1.0 | 0.8 | 0.7 | 0.9 | 0.3 | 0.0 | 0.8 | 0.7 | 0.9 | 1.1 | 1.0 | 0.8 | 1.2 | 1.0 | 0.9 | 1.1 | 0.9 | 0.9 | 0.8 | 1.0 | 0.9 | 1.2 | 0.9 | 0.9 | 1.2 | 1.4 | 1.3 | 0.8 | 0.8 | 1.2 | 0.7 | 1.0 | |
|  | **KICH** | 0.7 | 1.0 | 1.2 | 1.3 | 0.7 | 0.8 | 0.0 | 0.6 | 0.6 | 0.7 | 1.1 | 1.0 | 0.7 | 1.3 | 0.9 | 1.3 | 0.7 | 1.0 | 0.9 | 0.8 | 0.7 | 0.9 | 0.7 | 0.7 | 0.7 | 0.7 | 0.8 | 1.1 | 1.3 | 1.3 | 0.8 | 0.8 | |
|  | **KIRC** | 0.9 | 1.0 | 0.7 | 1.5 | 1.0 | 0.7 | 0.6 | 0.0 | 0.5 | 0.9 | 1.3 | 0.8 | 1.2 | 0.9 | 0.7 | 0.9 | 1.0 | 0.8 | 1.2 | 1.3 | 0.8 | 1.2 | 0.9 | 0.9 | 0.9 | 0.7 | 1.0 | 0.7 | 1.1 | 0.8 | 0.6 | 1.0 | |
|  | **KIRP** | 1.0 | 0.9 | 0.8 | 1.2 | 0.9 | 0.9 | 0.6 | 0.5 | 0.0 | 1.0 | 1.1 | 1.0 | 0.8 | 0.7 | 0.8 | 1.0 | 1.1 | 0.8 | 0.8 | 1.0 | 0.7 | 0.9 | 1.0 | 1.2 | 0.9 | 1.1 | 1.0 | 1.3 | 1.4 | 1.1 | 1.2 | 1.1 | |
|  | **BLCA** | 1.0 | 1.1 | 1.6 | 1.3 | 1.1 | 1.1 | 0.7 | 0.9 | 1.0 | 0.0 | 0.6 | 0.8 | 0.7 | 1.0 | 0.7 | 0.8 | 0.8 | 0.9 | 0.8 | 0.9 | 0.7 | 1.0 | 0.9 | 0.7 | 0.7 | 0.8 | 1.4 | 0.9 | 1.2 | 0.9 | 0.8 | 0.6 | |
|  | **CESC** | 0.8 | 1.2 | 1.2 | 0.9 | 0.6 | 1.0 | 1.1 | 1.3 | 1.1 | 0.6 | 0.0 | 0.7 | 0.7 | 0.9 | 0.7 | 1.0 | 1.2 | 1.2 | 1.1 | 0.8 | 0.8 | 0.8 | 1.0 | 0.8 | 0.8 | 1.3 | 1.5 | 1.2 | 1.0 | 0.8 | 0.9 | 1.0 | |
|  | **UCEC** | 0.8 | 0.8 | 0.8 | 0.9 | 0.9 | 0.8 | 1.0 | 0.8 | 1.0 | 0.8 | 0.7 | 0.0 | 0.6 | 0.9 | 0.8 | 1.1 | 0.9 | 0.9 | 1.4 | 1.4 | 0.9 | 1.3 | 1.1 | 1.3 | 0.9 | 1.0 | 1.2 | 0.7 | 1.2 | 0.8 | 0.7 | 1.3 | |
|  | **UCS** | 0.7 | 0.8 | 1.3 | 0.9 | 1.0 | 1.2 | 0.7 | 1.2 | 0.8 | 0.7 | 0.7 | 0.6 | 0.0 | 1.0 | 1.0 | 1.2 | 0.8 | 1.1 | 1.0 | 0.9 | 0.9 | 0.8 | 1.0 | 1.2 | 0.9 | 1.1 | 1.0 | 1.3 | 1.6 | 1.0 | 1.3 | 1.1 | |
|  | **COAD** | 1.2 | 1.1 | 0.9 | 0.9 | 1.1 | 1.0 | 1.3 | 0.9 | 0.7 | 1.0 | 0.9 | 0.9 | 1.0 | 0.0 | 0.3 | 0.7 | 1.0 | 0.8 | 1.0 | 1.3 | 1.5 | 0.9 | 0.8 | 1.1 | 1.0 | 1.2 | 1.3 | 1.3 | 1.4 | 1.0 | 1.3 | 1.4 | |
|  | **READ** | 0.9 | 1.3 | 1.0 | 1.1 | 0.9 | 0.9 | 0.9 | 0.7 | 0.8 | 0.7 | 0.7 | 0.8 | 1.0 | 0.3 | 0.0 | 0.7 | 0.8 | 0.9 | 1.2 | 1.2 | 1.4 | 1.0 | 0.7 | 0.7 | 0.8 | 1.0 | 1.4 | 1.3 | 1.3 | 1.0 | 0.9 | 1.3 | |
|  | **LAML** | 0.9 | 0.7 | 0.7 | 1.1 | 1.3 | 1.1 | 1.3 | 0.9 | 1.0 | 0.8 | 1.0 | 1.1 | 1.2 | 0.7 | 0.7 | 0.0 | 0.7 | 0.7 | 0.9 | 0.8 | 1.0 | 1.2 | 1.2 | 0.9 | 1.4 | 0.7 | 0.9 | 1.2 | 0.8 | 1.0 | 0.9 | 1.1 | |
|  | **ACC** | 0.8 | 0.8 | 1.0 | 0.9 | 1.0 | 0.9 | 0.7 | 1.0 | 1.1 | 0.8 | 1.2 | 0.9 | 0.8 | 1.0 | 0.8 | 0.7 | 0.0 | 0.6 | 0.8 | 0.9 | 1.1 | 1.4 | 1.0 | 0.7 | 1.1 | 0.8 | 0.8 | 1.1 | 1.1 | 1.6 | 1.1 | 1.2 | |
|  | **PCPG** | 0.9 | 0.9 | 0.7 | 0.9 | 1.3 | 0.9 | 1.0 | 0.8 | 0.8 | 0.9 | 1.2 | 0.9 | 1.1 | 0.8 | 0.9 | 0.7 | 0.6 | 0.0 | 1.0 | 0.8 | 0.9 | 1.3 | 1.4 | 0.9 | 1.0 | 1.0 | 1.1 | 0.8 | 0.8 | 1.2 | 1.3 | 1.3 | |
|  | **LUAD** | 1.4 | 0.9 | 1.4 | 0.9 | 0.9 | 0.8 | 0.9 | 1.2 | 0.8 | 0.8 | 1.1 | 1.4 | 1.0 | 1.0 | 1.2 | 0.9 | 0.8 | 1.0 | 0.0 | 0.7 | 0.8 | 0.8 | 1.0 | 1.0 | 1.0 | 1.2 | 1.0 | 1.2 | 1.0 | 1.3 | 1.2 | **0.6** | |
|  | **MESO** | 0.6 | 0.8 | 1.0 | 0.8 | 0.7 | 1.0 | 0.8 | 1.3 | 1.0 | 0.9 | 0.8 | 1.4 | 0.9 | 1.3 | 1.2 | 0.8 | 0.9 | 0.8 | 0.7 | 0.0 | 0.6 | 0.7 | 1.1 | 0.7 | 1.1 | 1.1 | 1.0 | 1.2 | 0.7 | 1.1 | 1.2 | 0.8 | |
|  | **THCA** | 0.9 | 0.8 | 1.0 | 1.3 | 0.7 | 0.9 | 0.7 | 0.8 | 0.7 | 0.7 | 0.8 | 0.9 | 0.9 | 1.5 | 1.4 | 1.0 | 1.1 | 0.9 | 0.8 | 0.6 | 0.0 | 1.1 | 1.4 | 1.0 | 0.8 | 0.9 | 0.9 | 0.9 | 0.7 | 0.9 | 0.8 | 0.6 | |
|  | **ESCA** | 0.9 | 1.2 | 1.2 | 0.9 | 0.9 | 1.2 | 0.9 | 1.2 | 0.9 | 1.0 | 0.8 | 1.3 | 0.8 | 0.9 | 1.0 | 1.2 | 1.4 | 1.3 | 0.8 | 0.7 | 1.1 | 0.0 | 0.5 | 0.8 | 0.8 | 1.1 | 1.0 | 1.1 | 1.1 | 0.7 | 1.2 | 0.7 | |
|  | **STAD** | 0.8 | 1.1 | 1.3 | 1.1 | 0.8 | 0.9 | 0.7 | 0.9 | 1.0 | 0.9 | 1.0 | 1.1 | 1.0 | 0.8 | 0.7 | 1.2 | 1.0 | 1.4 | 1.0 | 1.1 | 1.4 | 0.5 | 0.0 | 0.6 | 0.9 | 0.8 | 0.9 | 0.9 | 1.2 | 0.8 | 0.7 | 0.7 | |
|  | **PAAD** | 0.7 | 1.3 | 1.2 | 1.1 | 0.8 | 0.9 | 0.7 | 0.9 | 1.2 | 0.7 | 0.8 | 1.3 | 1.2 | 1.1 | 0.7 | 0.9 | 0.7 | 0.9 | 1.0 | 0.7 | 1.0 | 0.8 | 0.6 | 0.0 | 0.7 | 0.7 | 1.0 | 0.9 | 0.7 | 1.0 | 0.7 | 0.7 | |
|  | **PRAD** | 1.2 | 1.7 | 1.4 | 1.1 | 0.9 | 1.2 | 0.7 | 0.9 | 0.9 | 0.7 | 0.8 | 0.9 | 0.9 | 1.0 | 0.8 | 1.4 | 1.1 | 1.0 | 1.0 | 1.1 | 0.8 | 0.8 | 0.9 | 0.7 | 0.0 | 0.9 | 1.0 | 1.0 | 1.1 | 1.0 | 1.1 | 0.9 | |
|  | **GBM** | 0.8 | 1.0 | 1.1 | 1.4 | 1.4 | 1.4 | 0.7 | 0.7 | 1.1 | 0.8 | 1.3 | 1.0 | 1.1 | 1.2 | 1.0 | 0.7 | 0.8 | 1.0 | 1.2 | 1.1 | 0.9 | 1.1 | 0.8 | 0.7 | 0.9 | 0.0 | 0.4 | 0.8 | 0.9 | 0.7 | 0.6 | 0.7 | |
|  | **LGG** | 0.9 | 0.7 | 0.8 | 1.0 | 1.2 | 1.3 | 0.8 | 1.0 | 1.0 | 1.4 | 1.5 | 1.2 | 1.0 | 1.3 | 1.4 | 0.9 | 0.8 | 1.1 | 1.0 | 1.0 | 0.9 | 1.0 | 0.9 | 1.0 | 1.0 | 0.4 | 0.0 | 1.0 | 0.9 | 1.0 | 0.9 | 0.8 | |
|  | **TGCT** | 0.9 | 0.9 | 1.0 | 1.0 | 1.2 | 0.8 | 1.1 | 0.7 | 1.3 | 0.9 | 1.2 | 0.7 | 1.3 | 1.3 | 1.3 | 1.2 | 1.1 | 0.8 | 1.2 | 1.2 | 0.9 | 1.1 | 0.9 | 0.9 | 1.0 | 0.8 | 1.0 | 0.0 | 0.6 | 0.6 | 0.6 | 0.7 | |
|  | **THYM** | 0.9 | 0.9 | 0.7 | 0.8 | 0.9 | 0.8 | 1.3 | 1.1 | 1.4 | 1.2 | 1.0 | 1.2 | 1.6 | 1.4 | 1.3 | 0.8 | 1.1 | 0.8 | 1.0 | 0.7 | 0.7 | 1.1 | 1.2 | 0.7 | 1.1 | 0.9 | 0.9 | 0.6 | 0.0 | 0.8 | 0.7 | 0.7 | |
|  | **HNSC** | 0.9 | 1.0 | 1.0 | 1.2 | 1.3 | 1.2 | 1.3 | 0.8 | 1.1 | 0.9 | 0.8 | 0.8 | 1.0 | 1.0 | 1.0 | 1.0 | 1.6 | 1.2 | 1.3 | 1.1 | 0.9 | 0.7 | 0.8 | 1.0 | 1.0 | 0.7 | 1.0 | 0.6 | 0.8 | 0.0 | 0.7 | 0.7 | |
|  | **DLBC** | 0.7 | 0.8 | 1.0 | 1.4 | 0.7 | 0.7 | 0.8 | 0.6 | 1.2 | 0.8 | 0.9 | 0.7 | 1.3 | 1.3 | 0.9 | 0.9 | 1.1 | 1.3 | 1.2 | 1.2 | 0.8 | 1.2 | 0.7 | 0.7 | 1.1 | 0.6 | 0.9 | 0.6 | 0.7 | 0.7 | 0.0 | 0.6 | |
|  | **LUSC** | 1.1 | 1.0 | 1.5 | 1.3 | 1.0 | 1.0 | 0.8 | 1.0 | 1.1 | 0.6 | 1.0 | 1.3 | 1.1 | 1.4 | 1.3 | 1.1 | 1.2 | 1.3 | **0.6** | 0.8 | 0.6 | 0.7 | 0.7 | 0.7 | 0.9 | 0.7 | 0.8 | 0.7 | 0.7 | 0.7 | 0.6 | 0.0 | |

**Dataset Scaling and Comparison to Multi-layer Perceptron**

In addition to evaluating the disease subtypes, we sought to use the TCGA pan-cancer dataset to better elucidate the framework’s sensitivity to reductions in number of features and number of training samples. Classification performance was observed to increase linearly with number of training samples and logarithmically with available CpGs (Supplementary Figure 9). We also note that clustering performance from the VAE embeddings, as measured using the procedure outlined in the above section, generally increases with number of training samples and included features (Supplementary Figure 10). We also demonstrate comparable performance of training a multi-layer perceptron using similar training parameters and neural network architecture as *MethylNet’s* encoder-prediction structure (Supplementary Figure 9). *MethylNet’s* encoders have utility for generative and unsupervised tasks through capturing a low dimensional distribution of the data and the strengths in initializing the early layers of the prediction model with encoder pretraining.

**Supplementary Figure 8:** Micro F1-Scores of the held-out test samples (n=1676) of the TCGA cohort as they relate to: a) the fraction of training samples included for the training process, b) the number of CpGs. Test performance scales linearly with the number of training samples and logarithmically with the number of CpGs. Confidence intervals were calculated using a 1k nonparametric bootstrap of the test results for each dataset size point in the line plot, and the resulting bootstrapped f1-scores were used to compute the confidence interval for each point in the line plots; c) performance of MethylNet, pretrained using a VAE, is compared to performance using an MLP with the same architecture; F1-Score confidence intervals were derived using a 1k nonparametric bootstrap; validation loss for each model is compared at the first training epoch and their ultimate convergence point.

**Supplementary Figure 9:** V-Measure scores of the held-out test samples (n=1676) of the TCGA cohort as they relate to: a) the fraction of training samples included for the training process, b) the number of CpGs. V-measure scores were derived by applying and comparing hierarchical clustering on the VAE embeddings to known cancer subtype assignments and using a knee point detection algorithm to identify the ideal number of clusters for each tested dataset. In this figure, scores were smoothed using an exponential moving average smoothing technique to illustrate general trends.

**EWAS Analysis**

**Supplementary Figure 10:** Smoking EWAS study via MethylNet: a) final embeddings derived when finetuning the MethylNet VAE demonstrates cluster separation of the “never” versus “current” smokers; b) confusion matrix for the true and predicted “never” versus “current” smokers; c) plotted average ranks found using SHAP for the CpGs that intersected with CpGs identified by Liu et. al. versus the ranks of those corresponding p-values of the EWAS meta-analysis.

**Preliminary Results for PAM50 Classification of Breast Tumors**

In the main text, we described the results from our TCGA pan-cancer analysis, which are interesting but not too surprising given the cancers’ distinct differences in tissue of origin. Here, we have performed a preliminary analysis to discuss the ability to subtype within tissue and report preliminary results for the separation of the PAM50 molecular subtypes (Normal, Luminal A, Luminal B, HER2, Basal) for breast cancer. The data was acquired as per the acquisition and preprocessing procedures of (Titus, AJ, 2018), with data acquired from TCGA, and GEO accessions GSE84207, and GSE75067.

Supplementary Table 11: Preliminary Results for PAM50 Breast Tumor Classification (n=1,018); 95% confidence intervals of scores estimated via 1000-sample non-parametric bootstrap

| Dataset | Accuracy Score | F1-Score |
| --- | --- | --- |
| Train (n=712) | 0.99±0.0036 | 0.99±0.0035 |
| Validation (n=102) | 0.82±0.038 | 0.82±0.039 |
| Test (n=204) | 0.77±0.03 | 0.77±0.03 |

**Preliminary External Validation Cohort for Age Prediction**

We evaluated the potential for inclusion of external validation cohorts for use with our fit deep learning models. We chose to evaluate our age prediction model on an external cohort that contained many blood-based samples. We selected GEO accession GSE40279. Since the dataset only contained signal intensities and were not IDAT files, we preprocessed both GSE87571 and GSE40279 separately using BMIQ normalization instead of Noob normalization using *PyMethylProcess*. We utilized GSE87571 as our internal validation cohort (training, n=583, and validation sets, n=146), and GSE40279 (n=650) as our external validation cohort. We subselected 150,000 of the most variable CpGs from GSE87571. We chose to subselect a new set of CpGs and train a new model to avoid significant imputation that could confound the study when predicting on GSE40279 from a model fit on GSE87571. We fit a new prediction model on our internal validation cohort and evaluated the on the external validation cohort.

To evaluate performance of the model, we fit a linear model between the true and predicted ages for the training, validation and test sets using *scipy*. Our preliminary results indicate a strong fit on the external validation cohort ($\beta=1.06\pm0.02 SE,R^{2}=0.79$). Since we lacked ideal preprocessing quality control for GSE40279, and given that GSE40279 is fundamentally a different cohort than GSE87571 (Supplementary Figure 11a), and we only trained on one cohort, these results were to be expected. We expect that by training a deep learning age prediction model on more training cohorts (to represent a larger distribution of ages) and higher quality samples via more publicly available IDATs, external validation accuracy will continue to increase while we are able to include an expansive set of CpGs for inclusion in future studies.

**Supplementary Figure 11: Internal and External Validation Cohorts:** a) Boxenplot demonstrating distribution of ages for internal and external cohorts; notice how age for external validation cohort is greater than that of the internal validation cohort; b) plotted *MethylNet* predicted age versus actual age.

**Formulation of Variational Auto-Encoder**

Variational Auto-encoders (VAE) were used to extract biologically meaningful features for downstream prediction tasks. The major components of these auto-encoders are the encoder and decoder neural networks. The encoder finds a low dimensional vector that is truthful to the original sample and the decoder up-samples this vector into a close approximation of the original sample. The encoder is represented by a function $q_{\theta}$ with neural network parameters $\theta$, and acts on input $x$ to produce the hidden representation $q_{\theta}\left( z|x \right)$. The decoder, $p_{\phi}$, with neural network parameters $\phi$, transforms $z$ into $\hat{x}=p_{\phi}\left( x|z \right)$, an approximation of $x$. The neural network trains off of the loss function: $l_{Recon}\left( \theta,\phi\right)=l_{Recon}\left( x,p_{\phi}\left( {x|q}_{\theta}\left( z|x \right) \right) \right)$. This loss function is known as the reconstruction loss and measures the difference between the original and decompressed sample. The loss function used to specify this difference is usually binary cross entropy loss or mean squared error.

The goal of an auto-encoder is to learn a compressed representation of the data by compressing using the encoder and decompressing using the decoder as aforementioned. Variational auto-encoders are generative models that seek to generate new data by sampling from some underlying distribution to make the distances between the compressed data meaningful. Therefore, in addition to learning to accurately reconstruct the original data, it also tries model the parameters of the encoder $q_{\theta}\left( z|x \right)$ to learn this probability distribution, $p\left( z \right)$, a multivariate distribution typically assumed to be gaussian, but is an active area of research to find a better prior. To do this, regularization is added to the loss function specified for the vanilla auto-encoder:

$$l(\theta, \phi) = l_{Recon}\left( \theta,\phi\right) + KL(q_{\theta}\left( z|x \right)||p(z))$$

Where $KL$ represents the KL-Divergence, which measures the difference between the encoder distribution $q_{\theta}\left( z|x \right)$ and the assumed latent distribution of data $p\left( z \right)$. This function penalizes any encoder representation that diverges from the specified probability distribution. To increase the generative qualities of the VAE, the $KL$ is usually given more weight, $\beta$, to more heavily penalize divergences from the distribution at the expense of sacrificing the ability to more accurately reconstruct the original samples. The following loss function was implemented in MethylNet:

$$l\left( \theta,\phi\right)=l_{Recon}\left( \theta,\phi\right)-\beta*l_{KL}\left( \theta\right)$$

Where $l_{KL}\left( \theta\right)=- KL(q_{\theta}\left( z|x \right)||p(z))$. $\beta$ was set to 1 for the training of the analyses mentioned in the paper in order make the validation losses of different hyperparameter runs comparable and to reduce the weight given to data generation, but this is a modifiable hyperparameter that can be altered during the optimization of the model. The encoder $q_{\theta}\left( z|x \right)$ was used for both the embedding training and fine-tuning steps of MethylNet’s framework, but the decoder was discarded during prediction tasks. $p(z)$was assumed to be multivariate standard normal in the MethylNet framework. A reparameterization trick samples this distribution during both the training of the feature extractor and training of the downstream prediction layers during finetuning, which serves the purpose of sampling a new $z$ at every training step. This augmentation technique may make the final neural network more generalizable to new data. The reparameterization was shutoff during test time.

**Further Description of Transfer Learning Application**

Transfer learning describes the initial training of a neural network or model on one target of interest, which initializes the weights/parameters of the model so as to perform well on one task, then fine-tuning, or updating these learned weights on another task. The initial task to be performed is to derive embeddings of the DNA methylation profiles, which prepares the parameters of the model in such a way as to generate a meaningful latent space that is able to separate subtypes of interest, cellular proportions, and so forth without training on an explicit target. The fine-tuning or transfer learning of the network is the subsequent update of these model parameters to reflect training on the specific prediction task.

**More Information on SHAPley Attribution Method**

The SHAP (SHapley Additive ExPlanation) approach presents a possible framework to discover important CpGs for each prediction. They explain “black-box” models by simpler linear models. When applied to image analysis these methods translate the prediction of the model into heatmaps that overlay the original input image in a way that humans can understand. Applied to molecular profiles such as gene expression and CpG methylation information through interrogation of the coefficients of the simpler model, SHAP techniques can locate important genes and distinct CpGs that are drivers of association with an outcome variable.

The goal of Shapley feature attribution is to help the user understand why a machine learning model has made a specific prediction for any given sample. Many of these models are difficult to interpret. SHAP (Shapley Additive Explanations) is a model agnostic method to estimate the impact of each feature (assigned via Shapley values) on the prediction while remaining truthful to the properties of additivity, consistency and local accuracy.

Shapley values stem from game theory. A model’s output can be thought of as a reward to be distributed to a team of features that helped attain the reward. Shapley values dictate how the output of a model, the reward, should be shared amongst the features. Given the prediction of model $f$ on sample $x_{i}$ the method estimates shapley values $\phi\left( x_{i},f \right)$ by finding an approximation to the model, $g_{i}\left( x_{i} \right)$, one model per sample $x_{i}$. This model is said to be locally accurate: its output converges to $f\left( x_{i} \right)$ for this sample by summing up (additivity) $J$ attributions $\phi_{j}\left( x_{i},f \right)$ for $J$ features. It is also consistent: features that are truly important to one model’s predictions versus another are always assigned higher importance. To summarize these effects:

$$f\left( x_{i} \right)\approx g_{i}\left( x_{i} \right)=E\left[ f\left( x_{i} \right) \right]+\sum_{j=1}^{J} \phi_{j}\left( x_{i},f \right)$$

Where $E\left[ f\left( x_{i} \right) \right]$ is the expected reward across the training samples, which was missing albeit nonessential from the above explanation. These Shapley values can be calculated by a weighted sum of the contribution of the feature to a model’s prediction given all possible permutations of other features being introduced into the model. This method is computationally intractable, so various estimation methods for deriving the Shapley values have been implemented. MethylNet employs kernel, gradient-based, and DeepLift SHAP inference methods to make quick approximations of these Shapley values.

*The SHAPley analysis was employed in the manuscript to study aging and cellular heterogeneity, and smoking. Given that* $\phi_{j}$ *can be calculated for each individual, we can produce a matrix of shapley values that can be summed over:*

$$\{\phi_{ij}=\phi_{j}\left( x_{i},f \right)\}$$

*Then, we can calculate the Shapley values for each age group by summing the Shapley values across all of the individuals whose ages fall within the given ranges and arrive at aggregate measures for each age group k. A separate SHAP matrix is found for each cell-type, so an analogous task can be conducted by summing across all of the individuals for each cell type. Each age group was formed by binning the ages by discrete bin values (10 year increments), but Shapley values were calculated with respect to the prediction of a single individuals age and then averaged across the individuals of the group:*

$$\{\phi_{kj}=\left( \sum_{i\in\text{age group k}} \phi_{j}\left( x_{i},f \right) \right)/N_{k}\}$$

*Thus, the Shapley values were found by averaging attributions across* $N_{k}$ *individuals of the same group, which for cell types is the entire set of individuals.*

*In Figure 2c in the text, we rank the Shapley values of each age group from maximum to minimum using order statistics:*

$$\phi_{k}^{\left( j \right)}$$

*Where (j) here is the jth largest Shapley value corresponding its respective feature and then look for the number of Hannum CpGs that overlap with the set of the top 1000* $\phi_{k}^{\left( j \right)}$*.*

*As for Figure 2d, we calculate the correlation distance between the Shapley values between pairs of age groups (i and jth age groups) as such:*

$$d_{\mathrm{ij}}=d\left( \boldsymbol{\phi}_{\boldsymbol{k}_{\boldsymbol{i}}}\boldsymbol{,}\boldsymbol{\phi}_{\boldsymbol{k}_{\boldsymbol{j}}} \right)=1-\frac{\left( \boldsymbol{\phi}_{\boldsymbol{k}_{\boldsymbol{i}}}-\bar{\boldsymbol{\phi}_{\boldsymbol{k}_{\boldsymbol{i}}}} \right)\cdot\left( \boldsymbol{\phi}_{\boldsymbol{k}_{\boldsymbol{j}}}-\bar{\boldsymbol{\phi}_{\boldsymbol{k}_{\boldsymbol{j}}}} \right)}{\left| \left| \left( \boldsymbol{\phi}_{\boldsymbol{k}_{\boldsymbol{i}}}-\bar{\boldsymbol{\phi}_{\boldsymbol{k}_{\boldsymbol{i}}}} \right) \right| \right|_{2}\left| \left| \left( \boldsymbol{\phi}_{\boldsymbol{k}_{\boldsymbol{j}}}-\bar{\boldsymbol{\phi}_{\boldsymbol{k}_{\boldsymbol{j}}}} \right) \right| \right|_{2}}$$

*Where* $\boldsymbol{\phi}_{\boldsymbol{k}_{\boldsymbol{i}}}$ *is the sequence of shapley scores in the ith age group. Since due to high collinearity, it can be difficult to pinpoint exactly which CpGs are important in each age group, the correlation between the two age groups suggests that the two age groups have a similar overall profile of important CpGs that are associated with their outcome. Thus, clustering of these distances can yield relationships between the age groups that can corroborate with what we would expect in reality, and a similar analysis was conducted for cell-types in Figure 3c.*

*For the smoking comparison, we selected CpGs in our SHAP array that corresponded to the CpGs used in the cigarette smoking study, and then rank ordered the absolute value of each of the individual’s CpG SHAP scores. We averaged the ranks of the CpGs across the individuals to ascertain the overall importance of the CpGs to compare to the ordering found from the traditional EWAS analysis.*

**Example Code to Run Pipeline**

Example code for running the pipeline can be found at <https://github.com/Christensen-Lab-Dartmouth/MethylNet/tree/master/example_scripts>. Further instructions can be found in the README and wiki page.

Here, we have added a few simple commands to run *MethylNet*:

Preparing the DNAm data and splitting into training, validation and test sets:

*pymethyl-utils methy_array_from_csv -beta beta.csv -pheno pheno.csv*

*pymethyl-utils train_test_val_split -tp .8 -vp .125*

Train a model to embed DNAm data using a VAE:

*methylnet-embed launch_hyperparameter_scan -cu -sc Age -j 20*

*methylnet-embed launch_hyperparameter_scan -cu -sc Age -n 1*

Train a model to predict age after training the VAE:

*methylnet-predict launch_hyperparameter_scan -cu -ic Age -j 20*

*methylnet-predict launch_hyperparameter_scan -cu -ic Age -n 1*

The code can also be run on Code Ocean without install at: [https://doi.org/10.24433/CO.6373790.v1](https://nam12.safelinks.protection.outlook.com/?url=https%3A%2F%2Fdoi.org%2F10.24433%2FCO.6373790.v1&data=02%7C01%7CJoshua.J.Levy.GR%40dartmouth.edu%7C718c68856cfb4061228f08d78255538a%7C995b093648d640e5a31ebf689ec9446f%7C0%7C0%7C637121175303952242&sdata=Wk%2F2494rKtM3BGlOYNy2a6Wakz10LQwzRme8%2B9rYH0I%3D&reserved=0) .

**Hyperparameter Scan Details for Embedding and Prediction Tasks**

A robust hyperparameter scan was conducted for both the embedding and prediction tasks. A list of a user supplied search grid could be supplied; MethylNet also has its own built in search options. Typically, a number of randomized searches are made, the lowest validation losses from each of these runs are returned. Then, the top job(s) can be rerun to try to find the set of parameters with the lowest loss. Rerunning the best performing jobs may not yield the lowest validation loss due to the stochastic nature of initializing the neural network parameters and the samples shuffled during the training iterations, so it is recommended to rerun the analyses with the same set of hyperparameters multiple times. The following are a list of select hyperparameters that could be modified for these tasks:

- Number of epochs to train for
- Number of Latent Dimensions (Embedding Tasks only)
- Learning Rate (during prediction tasks, one is specified for feature extractor, the other for the fine-tuning layers)
- Beta (Embedding Tasks, weight given to KL-Loss)
- KL warm up (Embedding Task, number of epochs to apply lower weight to KL Loss to focus on reducing reconstruction loss)
- Scheduler (function that modulates the learning rate; for instance, interrupted cosine or horizontal line)
- Parameters relating to scaling of interrupted cosine learning rate scheduler
- Batch size
- Model Complexity (coefficient that determines how wide and deep neural network should be) and hidden layer topology (the topology of the encoder is mirrored to form the decoder)

Below are the top 3 unique sets of select hyperparameters for MethylNet’s embedding and prediction tasks selected for the age estimation, cell-type deconvolution and pan-cancer classification analyses:

Supplementary Table 12: Select Hyperparameters for Embedding Tasks

| Task | Number Epochs | Best Epoch | Minimum Val Loss | Number Latent Dimensions | Encoder Hidden Layer Topology | Learning Rate | Batch Size |
| --- | --- | --- | --- | --- | --- | --- | --- |
| Age Estimation | 500 | 306 | 9159250 | 150 | [100, 200, 300] | 0.01 | 50 |
|  | 200 | 40 | 9181492 | 100 | [500, 100] | 0.05 | 50 |
|  | 200 | 193 | 9196771 | 100 | [500, 100] | 0.01 | 50 |
|  |  |  |  |  |  |  |  |
| Cell-Type Deconvolution | 500 | 246 | 9157337 | 100 | [300] | 0.005 | 50 |
|  | 100 | 98 | 9160540 | 100 | [] | 0.01 | 50 |
|  | 700 | 499 | 9169060 | 300 | [200, 1000, 100] | 0.1 | 50 |
|  |  |  |  |  |  |  |  |
| Pan-Cancer | 700 | 510 | 88319693 | 150 | [200, 100, 200] | 0.001 | 512 |
|  | 500 | 305 | 88436906 | 100 | [500, 100] | 0.005 | 512 |
|  | 700 | 636 | 88526436 | 100 | [300, 200] | 0.0005 | 256 |

Supplementary Table 13: Select Hyperparameters for Prediction Tasks

| Task | Number Epochs | Best Epoch | Minimum Validation Loss | Fine-Tuning Layer Topology | Learning Rate Feature Extractor | Learning Rate Fine-tuning Layers | Batch Size | Dropout |
| --- | --- | --- | --- | --- | --- | --- | --- | --- |
| Age Estimation | 700 | 482 | 828.46 | [200] | 0.1 | 0.5 | 256 | 0 |
|  | 200 | 173 | 942.90 | [] | 0.1 | 0.0001 | 100 | 0.2 |
|  | 200 | 148 | 1029.74 | [100, 100, 200] | 0.1 | 0.0001 | 100 | 0 |
|  |  |  |  |  |  |  |  |  |
| Cell-Type Deconvolution | 700 | 630 | 0.12 | [1000] | 0.05 | 5E-05 | 256 | 0.1 |
|  | 500 | 485 | 0.18 | [200, 500] | 0.1 | 5E-05 | 50 | 0 |
|  | 500 | 479 | 0.24 | [500, 1000] | 0.0005 | 0.005 | 500 | 0 |
|  |  |  |  |  |  |  |  |  |
| Pan-Cancer | 200 | 126 | 2.19 | [300] | 0.001 | 1E-05 | 50 | 0 |
|  | 500 | 256 | 2.22 | [] | 5E-05 | 0.0005 | 50 | 0 |
|  | 500 | 204 | 2.31 | [200] | 0.001 | 5E-05 | 50 | 0.2 |

More information on the hyperparameter scans can be found in the supplied GitHub repository.

**MethylNet Execution Time**

The execution time of hyperparameter scans can be dependent on the solver being used, the specified search grid, size of the dataset, and hardware specifications of the compute instance or the scheduling system of a compute cluster (eg. controlling the number of jobs that can execute in parallel). In addition, peak performance, or an acceptable level of performance, can be stochastically achieved within the first few iterations of the scan or execution of the algorithm. As such, this can make it difficult to estimate how long the entire workflow takes to execute. Our test demo, located on Code Ocean here: <https://doi.org/10.24433/CO.6373790.v1> , takes 20-25 minutes to run, processing the hyperparameter scan on the order of 50 sequential executed jobs. We have noticed when scaling to thousands of jobs it took only a few hours to finish computation. As an example of the execution time for a single task, we noticed that training a prediction job on the 9,500 individuals from the TCGA cohort with 200,000 CpGs for 50 epochs, including the time it took to generate visualizations, took 11 minutes on a Nvidia Tesla K80 GPU.

**Hyperparameter Scan Details for SVM Tasks**

To compare MethylNet’s Pan-Cancer predictions to another leading machine learning technique, a robust hyperparameter scan was conducted to find the best set of hyperparameters for an L2-penalized support vector machine model. Similar to MethylNet, the set of hyperparameters with the highest validation f1-score was chosen; class weights were used to account for any class imbalances. Listed below are the model’s hyperparameters and their range:

- Kernel: linear or radial basis (RBF) decision functions
- Inverse weight given to L2-penalty (high value has low regularization): 1, 10, 100, 1000
- Gamma (influence of training sample for RBF kernel): 1, 0.1, 0.001, 0.0001

A randomized grid search that tested 24 sets of hyperparameters was conducted to find the best set of hyperparameters. This model was trained and tested using this set to produce the results from the paper.

**Embedding Visualizations for Ages and Cell-Type Proportions**

**Supplementary Figure 12:** Fine-tuned embeddings (few parts highlighted) for: a) Age Prediction, b) Cell-Type Deconvolution (colored by Neutrophil Cell Type Proportions), and c) Pan-Cancer Classification (Labeled kidney cancers, lung cancers, brain cancers)

Because of difficulties in interpretation associated with the distortion of perspective and UMAP parameters, these embeddings are also supplied as interactive three-dimensional plots and have been included in the GitHub repository at the following URL: <https://github.com/Christensen-Lab-Dartmouth/MethylNet/tree/master/methylnet_results/embeddings>

**Model Training Information and Training Curves**

Binary Cross Entropy Loss was used as the loss function for the regression analyses, and Cross Entropy Loss was used for the classification approach. At every hidden layer in the network, non-linear transforms were applied via the ReLU transform, and during training, some of the neurons of these network layers were deactivated via Dropout for improved generalizability. The network was specified to use a sigmoid function to restrict the output of the regression tasks between 0 and 1. Throughout the training iterations, the learning rate was modulated using an interrupted cosine function to best capture trade-offs between exploration and exploitation of the objective. This functionality was sometimes removed at various points during the hyperparameter scans. The model with the lowest validation loss at a particular training iteration was returned for each run. The models were trained using a Nvidia Tesla K80 Graphics Processing Units, accessed via the Dartmouth Discovery Research Computing compute cluster.

Plotted below are the training curves of the auto-encoding and fine-tuning/transfer learning steps:

**Supplementary Figure 13: Model Training Curves for** a) Age Predictions, b) Cell-Type Predictions, c) Pan-Cancer Predictions. Please note that the learning rates for the prediction curve of a) oscillates quickly every 10 training epochs as compared to a larger timescale.
